# Academic Library Metamorphosis and Regeneration

**DOI:** 10.5195/jmla.2019.600

**Published:** 2019-01-01

**Authors:** Charlotte Beyer

**Affiliations:** Boxer Library, Rosalind Franklin University of Medicine and Science, North Chicago, IL, charlotte.beyer@rosalindfranklin.edu

In 2014, information studies honor society Beta Phi Mu partnered with Rowman & Littlefield to publish a series of titles that inspire thoughtful conversations in the field of librarianship, while supporting the society’s commitment to scholarship, leadership, and service*. Academic Library Metamorphosis and Regeneration* by Marcy Simons is the sixth title in this series and is focused on documenting the history of change in libraries, while also looking to the future.

At only 131 pages, this work is a fairly light read but provides an adequate summary of how general academic libraries have transformed in the past fifty years. Readers should be advised that while it is a good overall summary of change, this volume does not discuss specific changes and transformations in academic health sciences librarianship at all. Readers who are specifically interested in health sciences librarianship would be better served by reviewing other Rowman & Littlefield titles such as *Health Sciences Librarianship* (2014; ISBN: 978-0-8108-8812-8), edited by M. Sandra Wood, FMLA; or *Transforming Medical Library Staff for the Twenty-First Century* (2017; ISBN: 978-1-4422-7219-4), edited by Melanie J. Norton and Nathan Rupp.

Simons’s book is divided into eight chapters, beginning with the environmental factors that inspired change, continuing with topics such as changing roles and change management, and then ending with an annotated bibliography of resources for further exploration into the topic of library transformation and change. The content flows well due to the numerous citations of important articles and projects in the field of academic librarianship.

Also, the chapters feel interconnected, with information in one chapter leading to another. For example, in the “Declines in Reference” section, located in the first chapter, “Sea of Change,” Simons describes factors that led to reduction of traditional reference questions and then informs readers that the topic of transformations for reference librarians is highlighted in the chapter, “Changes in Traditional Roles.” This transition was very effective in keeping my interest in the content fresh. The chapter content is also broken down into multiple small sections that are easily digestible, making it a quick read.

One useful portion of this book is a chapter dedicated to two annotated bibliographies. The first bibliography relates to change in libraries and organizational development, and the other focuses on general information on change and transformation. These entries provide readers with opportunities for further exploration into these topics, so learning about change and transformation in libraries does not stop with the final page.

This work effectively summarizes the history of changes in general academic librarianship, while looking forward. In other words, it identifies that where the profession is going is influenced by where it has been. Librarians who are seeking a light introduction into transformation in general academic libraries should read this book. This book would be especially helpful for new librarians who want an introduction to how libraries have evolved over the previous decades. Those wanting a health sciences librarianship focus should examine the titles mentioned earlier.

